# A Coumarin-Based Fluorescent Probe for Ratiometric Detection of Cu^2+^ and Its Application in Bioimaging

**DOI:** 10.3389/fchem.2020.00800

**Published:** 2020-10-02

**Authors:** Jie Zhang, Meng-Yu Chen, Cui-Bing Bai, Rui Qiao, Biao Wei, Lin Zhang, Rui-Qian Li, Chang-Qing Qu

**Affiliations:** ^1^School of Chemistry and Materials Engineering, Fuyang Normal University, Fuyang, China; ^2^Key Laboratory of Photochemical Conversion and Optoelectronic Materials, Technical Institute of Physics and Chemistry of the Chinese Academy of Sciences (TIPC-CAS), Beijing, China; ^3^Engineering Research Center of Biomass Conversion and Pollution Prevention of Anhui Educational Institutions, Fuyang, China; ^4^Research Center of Anti-aging Chinese Herbal Medicine of Anhui Province, Fuyang, China

**Keywords:** coumarin, fluorescent probe, Cu^2+^, test strips, bioimaging

## Abstract

The fluorescent probe **L**, based on naphthalimide-modified coumarin, was designed, synthesized, and characterized, which could recognize Cu^2+^ from other cations selectively and sensitively in HEPES buffer (10 mM, Ph = 7. 4)/CH_3_CN (1:4, V/V). When the probe **L** interacted with Cu^2+^, the color and the fluorescent intensity changed obviously and it provided the naked-eye detection for Cu^2+^. The recognition mode between them was achieved by Job's plot, IR, MS, SEM, and ^1^HNMR. In addition, test strips made from **L** could still interact with Cu^2+^ in tap water effectively. The limit of detection (LOD) of **L** was 3.5 × 10^−6^ M. Additionally, the density functional theory (DFT) calculation method was used to analyze the action mechanism of **L** toward Cu^2+^. Importantly, the fluorescent probe **L** could demonstrate favorable selectivity toward Cu^2+^ in *Caenorhabditis elegans*. Thus, **L** was considered to have some potential for application in bioimaging.

## Introduction

As is known to all, copper ion (Cu^2+^) occupies an important place in a variety of fundamental physiological processes in organisms ranging from bacteria to mammals (Huang et al., 2014; Arjmand et al., 2018; Wang P. et al., 2018; Wang Y. et al., 2018; Zeng et al., 2019; Aydin et al., 2020; Wang Z. G. et al., 2020). However, Cu^2+^ can also lead to environmental pollution because it has been used widely in industrial and agricultural processes (Huang et al., 2014; Zhang et al., 2016; Gu et al., 2018; Wang et al., 2019; Zhu et al., 2020). So, it is urgent to develop some methods for recognition toward Cu^2+^. Owing to their advantage over other analytical methods which include atomic absorption spectrometer (ABS) and inductively coupled plasma mass spectrometry (ICP-MS), fluorescent probes have received more and more attention in the past few decades (Ge et al., 2013; Chen et al., 2017; Han et al., 2017; Wang et al., 2017; Zhou et al., 2017; Liu et al., 2018; Tian et al., 2019; Pipattanawarothai and Trakulsujaritchok, 2020). Therefore, various kinds of probes have been reported, such as rhodamines (Zhang et al., 2020), phenanthroline (Nawaz et al., 2018), anthracene (Shree et al., 2019), coumarin (Qi et al., 2018; Qu et al., 2019; Zhu et al., 2020), and BODIPY (Cetinkaya et al., 2019; Fang et al., 2019; Xia et al., 2019). In contrast with other derivatives, coumarin derivatives represent good fluorescence properties, excellent photostability, and easy preparation (Zhang et al., 2014; Hossain et al., 2017; Kumari et al., 2017). Consequently, many coumarin derivatives have been obtained to detect Cu^2+^ (Li et al., 2018; Roy et al., 2018; Wang Y. et al., 2018; Zhao et al., 2019; Joniak et al., 2020).

Formylcoumarins have been linked with aromatic amine through C=N to acquire the derivatives (Qin et al., 2016; He et al., 2019; Srivastava et al., 2019). Although few derivatives, which are connected by amide linkage, have been reported. It is obvious that the amide-modified derivatives have more potential sites to interact with Cu^2+^ by amide than by C=N, which might enhance their selectivity and sensitivity (Bai et al., 2019).

**Scheme 1:**
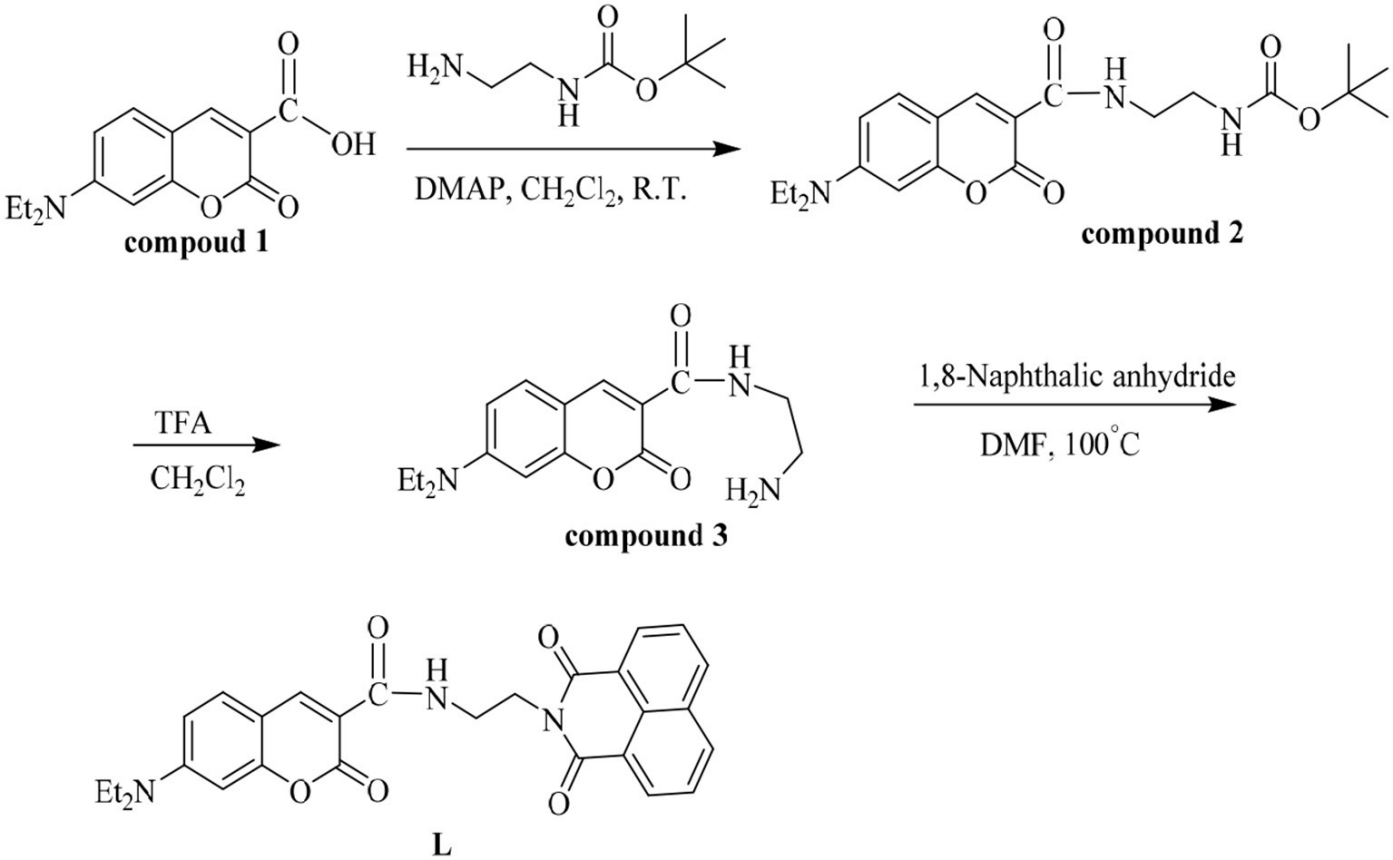
Synthesis of probe **L**.

In this paper, a naphthalimide-modified probe **L** (Scheme 1) based on coumarin was designed and synthesized with amide. It is interesting that probe **L** could distinguish Cu^2+^ from other cations selectively and sensitively in HEPES buffer (10 mM, pH = 7.4)/CH_3_CN (1:4, V/V) observable by the naked-eye. In addition, test strips made from **L** could also detect Cu^2+^ successfully. Importantly, probe **L** could identify Cu^2+^ in *Caenorhabditis elegans*. From these data, probe **L** has potential applications in bioimaging.

## Experimental

### Instruments and Reagents

^1^H NMR and ^13^C NMR spectra were both performed on a Bruker at 400 MHz using TMS as an internal standard (DMSO-*d*_6_ as the solvents). Infrared spectra were obtained on a Nicolete 5700 FT-IR spectrophotometer. Mass spectra were carried on with a Shimadzu LCMS-IT/TOF mass spectrometer. UV-Vis absorption spectra were studied on a Shimadzu UV-1601 spectrophotometer. Fluorescence spectrum was operated on a HORIBA FLUOROMAX-4-NIR spectrometer. Biological imaging was performed on a LEICA DM 2500. All reagents used were of analytical grade.

### Synthesis of Probe L

Compounds **1-3** were gained according to the previous work (Ma et al., 2004; Yu et al., 2011; Tanaka et al., 2019; Wang P. et al., 2020; Wang Z. G. et al., 2020). Then compound **3** (1 mmol, 304 mg) and 1.8-naphthalic anhydride (1 mmol, 198 mg) were dissolved in 25 mL DMF and heated to reflux for 6 h. After the reaction was complete, the reaction mixture was cooled to room temperature and poured into ice water to separate the solid. The solid was purified by silica gel chromatography (methylene chloride: methanol=30:1) to obtain probe **L** (Yield 75%). ^1^H NMR (400 MHz, DMSO-*d*_6_) δ 8.77 (t, J = 6.0 Hz, 1H), 8.52 (s, 1H), 8.44 (t, J = 8.3 Hz, 4H), 7.84 (t, J = 7.7 Hz, 2H), 7.60 (d, J = 9.0 Hz, 1H), 6.79 – 6.72 (m, 1H), 6.62 – 6.53 (m, 1H), 4.28 (t, J = 5.7 Hz, 2H), 3.66 (q, J = 5.9 Hz, 2H), 3.46 (q, J = 7.1 Hz, 4H), 1.32 – 1.20 (m, 1H), 1.12 (t, J = 7.0 Hz, 6H), 1.06 (s,1 H) (Supplementary Figure 1). ^13^C NMR (100 MHz, DMSO-*d*_6_) δ 164.12, 163.13, 161.75, 157.61, 152.81, 148.02, 134.64, 131.97, 131.76, 131.09, 127.96, 127.65, 122.71, 110.50, 109.91,107.99, 96.27, 44.78, 37.92, 12.77 (Supplementary Figure 2). HRMS (ESI) m/z: [**L+**H]^.+^ Calcd for C_28_H_26_N_3_${\text{O}}_{5}^{+}$: 484.19; Found 484.15 (Supplementary Figure 3a).

### General Spectroscopic Method

Solutions of metal ions were prepared from their nitrates salts of K^+^, Fe^2+^, Ca^2+^, Na^+^, Ag^+^, Cu^2+^, Co^2+^, Mg^2+^, Cd^2+^, Ni^2+^, Ba^2+^, Pb^2+^, Al^3+^, Sr^2+^, Mn^2+^, Zn^2+^, Hg^2+^, Ce^3+^, Y^3+^, and Fe^3+^. The ligand concentration (**L**) was kept constantly at (1.0 × 10^−5^ M). The solution of the probe was prepared in HEPES buffer (10 mM, pH = 7.4)/CH_3_CN (1:4, V/V).

## Results and Discussion

### Study on Spectral Properties of the Probe L

**Figure 1:**
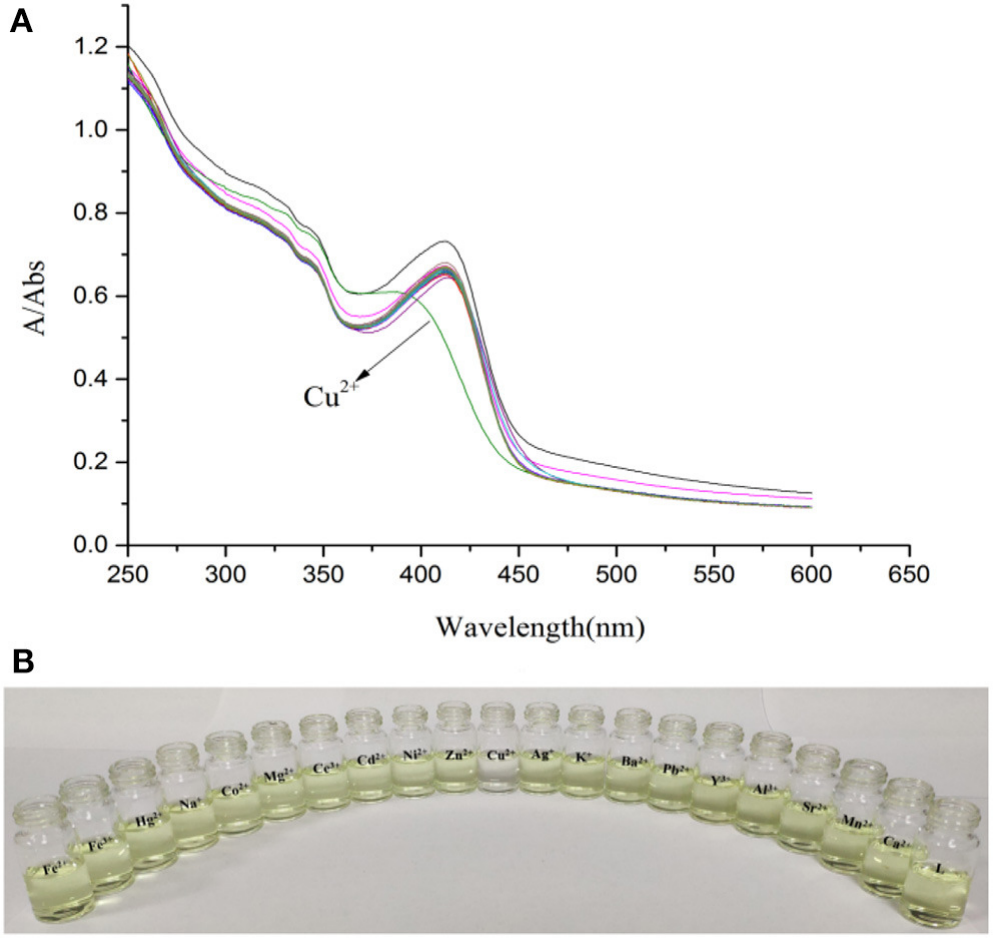
**(A)** Absorption spectra of **L** (1.0 × 10^−5^ M) in the presence of various metal ions K^+^, Na^+^, Ag^+^, Cu^2+^, Co^2+^, Ca^2+^, Cd^2+^, Mg^2+^, Ba^2+^, Pb^2+^, Sr^2+^, Fe^2+^, Ni^2+^, Zn^2+^, Mn^2+^, Hg^2+^, Al^3+^, Y^3+^, Ce^3+^, and Fe^3+^ (3.0 × 10^−5^ M) in HEPES buffer (10 mM, pH = 7.4)/CH_3_CN (1:4, V/V). **(B)** Photograph of **L** (1.0 × 10^−5^ M) in the presence of various metal ions (3.0 × 10^−5^ M) in HEPES buffer (10 mM, pH = 7.4)/CH_3_CN (1:4, V/V).

**Figure 2:**
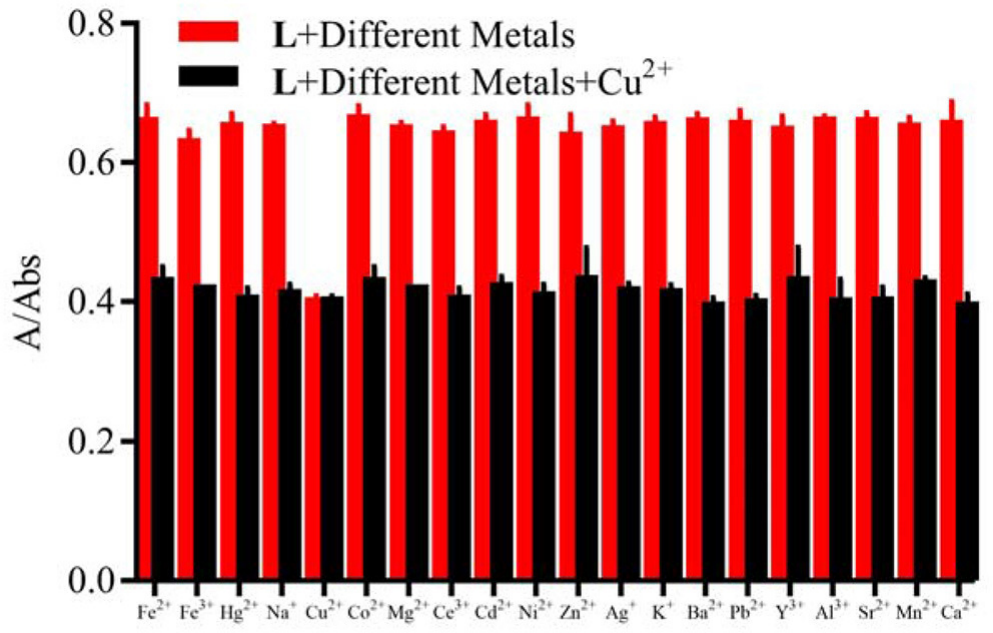
Competitive selectivity of **L** (1.0 × 10^−5^ M) toward Cu^2+^ (3.0 × 10^−5^ M) in the presence of other metal ions (3.0 × 10^−5^ M) in HEPES buffer (10 mM, pH = 7.4)/CH_3_CN (1:4, V/V), the absorbance at 412 nm.

Physiological pH (e.g., in the human body) is between 7.35 and 7.45 (Lee et al., 2010), thus, pH 7.4 was used in the subsequent study, in which Cu^2+^ in adult *C. elegans* was detected. The effect of pH on the fluorescent signal was investigated (Supplementary Figure 10). When the solution of Cu^2+^ was added into **the L** solution, the maximum absorption peak shifted from 412 nm to 385 nm (Figure 1A). The solution color changed from faint yellow to colorless (Figure 1B). While other cations didn't cause any change. It was clear from the competitive experiment that other cations have little impact on the selectivity of **L** toward Cu^2+^ (Figure 2). According to UV-Vis spectroscopy, the **L** solution color change caused by Cu^2+^ could be observed directly by the naked-eye. So, the fluorescent probe **L** can demonstrate favorable selectivity toward Cu^2+^among other metals.

**Figure 3:**
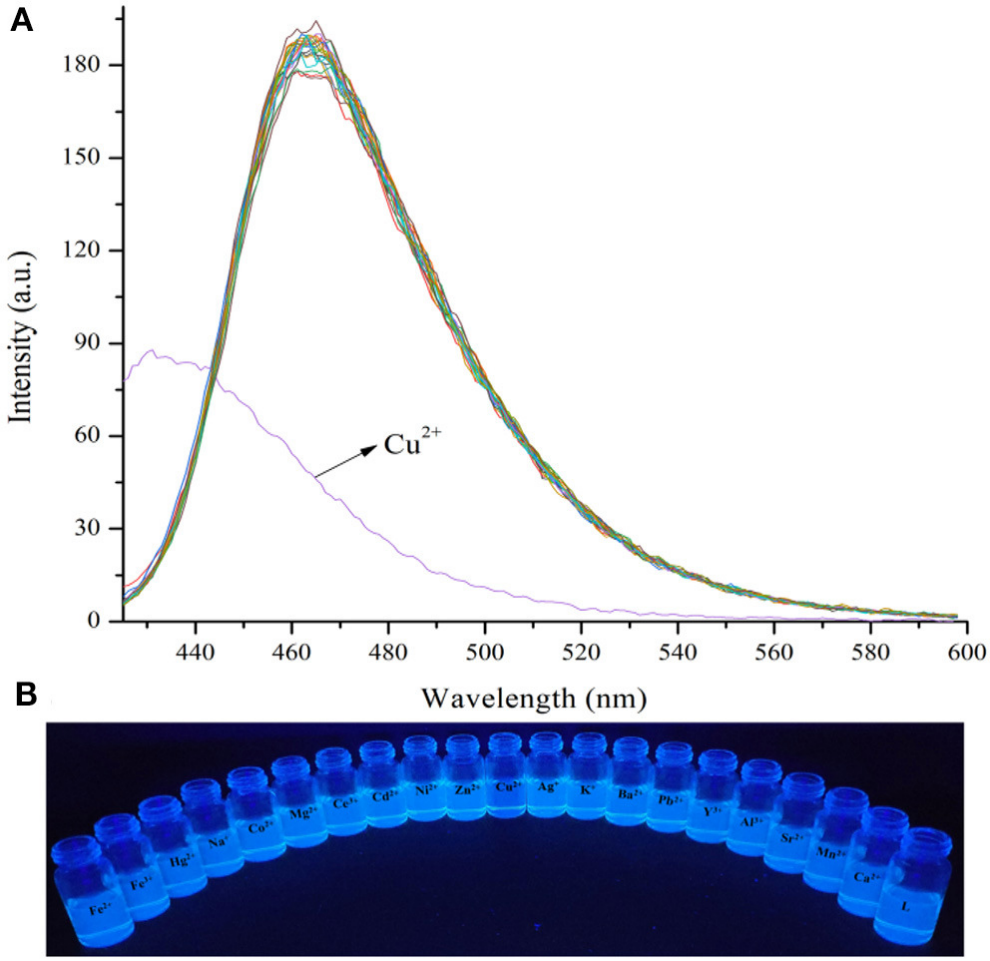
**(A)** Fluorescence spectra changes of **L** (1.0 × 10^−5^ M) in the presence of various metal ions (3.0 × 10^−5^ M) in HEPES buffer (10 mM, pH = 7.4)/CH_3_CN (1:4, V/V), λ_ex_ =412 nm, detection from 425 to 600 nm. **(B)** Photograph of probe **L** (1.0 × 10^−5^ M) in the presence of various metal ions (3.0 × 10^−5^ M) in HEPES buffer (10 mM, pH = 7.4)/CH_3_CN (1:4, V/V) under a 365 nm UV lamp.

**Figure 4:**
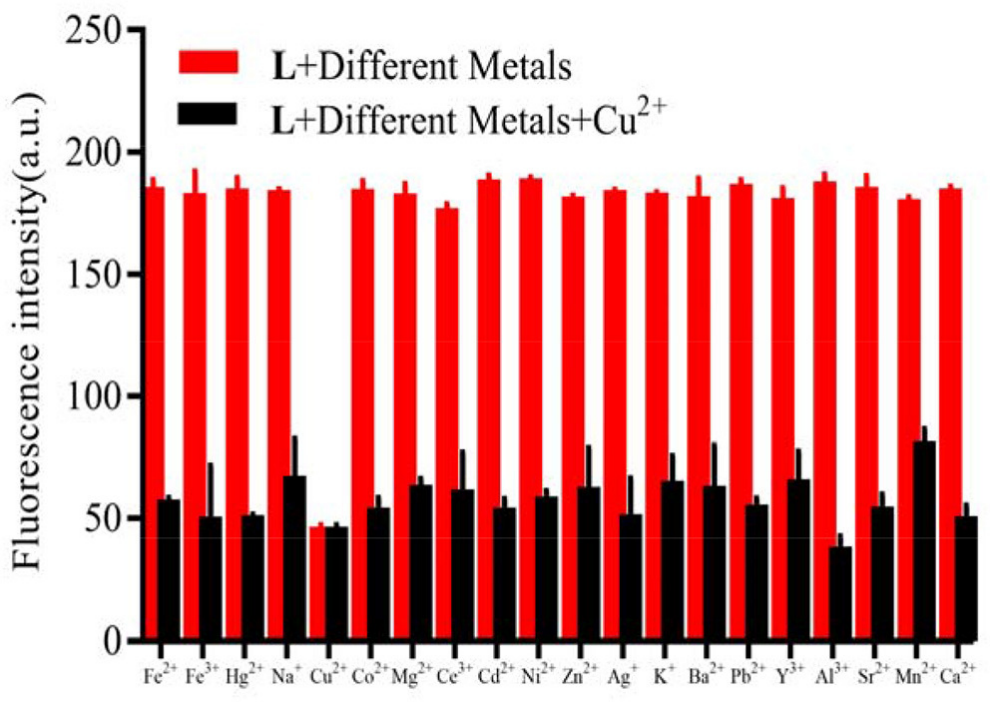
Competitive selectivity of **L** (1.0 × 10^−5^ M) toward Cu^2+^ in the presence of other metals (3.0 × 10^−5^ M) in HEPES buffer (10 mM, pH = 7.4)/CH_3_CN (1:4, V/V), λ_ex_ = 412 nm, the fluorescent intensity at 465 nm.

When the probe **L** was excited by 412 nm, the fluorescent emission peak appeared at 465 nm. Interestingly, only Cu^2+^ caused the fluorescent intensity at 465 nm to reduce when the cation solution was added (Figure 3A). The fluorescent change induced by Cu^2+^ could also be observed easily by the naked-eye under a 365 nm UV lamp (Figure 3B). According to the competition experiment, other cations seldom interfered with the detection of **L** toward Cu^2+^ (Figure 4). Moreover, the limit of detection for **L** toward Cu^2+^ was calculated to be 3.5 × 10^−6^ M (Supplementary Figure 8). Based on the data above, it was concluded that **L** might recognize Cu^2+^ selectively and sensitively.

### The Interaction Mode Between L and Cu^2+^

**Figure 5:**
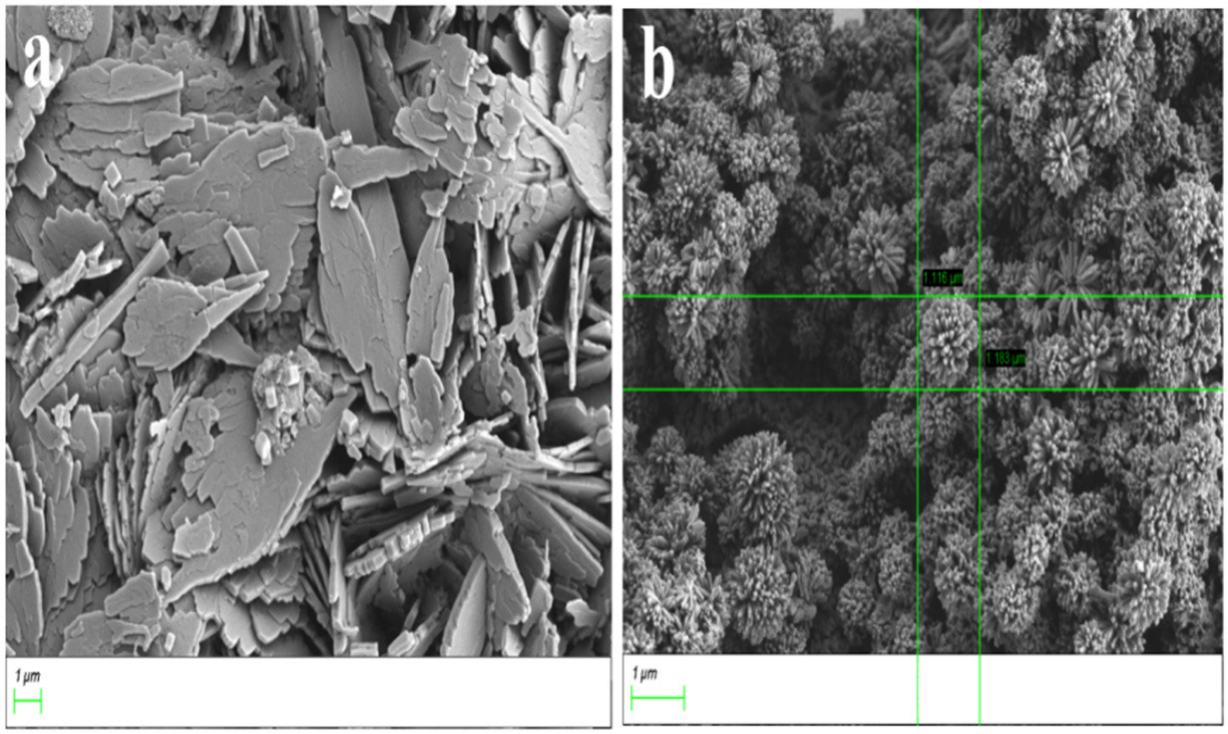
SEM micrographs of **L** and **L**-Cu^2+^. **(a)** SEM micrographs of **L**; **(b)** SEM micrographs of **L**-Cu^2+^.

In order to determine the stoichiometric ratio of **L** toward Cu^2+^, the molar method (Supplementary Figure 4) and the continuous variation method (Supplementary Figure 5) were both carried out. The results showed that the stoichiometric ratio was 1:1 between them. To our great joy, the result was supported by mass spectral analyses because the ion peak was detected at m/z 670.08 which was in accordance with [**L**+Cu^2+^+${2\text{NO}}_{3}^{-}$] ^+^ (Supplementary Figure 3b). On the basis of the data, it was concluded that the stoichiometric ratio between them was 1:1 when **L** interacted with Cu^2+^. To study how Cu^2+^ changed the **L** aggregation morphology, a SEM experiment was performed. When **L** (1 equiv**)** combined with the Cu^2+^(2 equiv), it was discovered that the **L** morphology changed from the layer to the petal shape whose diameter was 1 um (Figure 5) which may be the result of the interaction between the fluorescent probe **L** and Cu^2+^.

**Figure 6:**
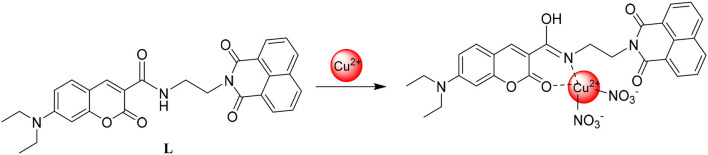
The interaction mode between **L** and Cu^2+^.

To clarify the interaction mode between **L** and Cu^2+^, IR analyses and ^1^H NMR titration were conducted. From IR (Supplementary Figure 6), the absorption band at 1,704 cm^−1^ assigned to the C=O stretching vibration vanished when **L** (1 equiv) interacted with Cu^2+^ (2 equiv). The absorption band at 3,337 cm^−1^ assigned to the N-H stretching vibration also disappeared, were the absorption band at 1,537 cm^−1^ corresponding to the stretching vibration of C=N appeared. The amide group tautomerized to C=N once **L** associated with Cu^2+^. It is important that the conclusion from IR was in accordance with the ^1^H NMR titration. As the Cu^2+^ concentration increased, the chemical shift of N-H in the amide group at 8.50 disappeared by a degree (Supplementary Figure 7). From the above data, the interaction mode between **L** and Cu^2+^ was shown as (Figure 6).

### Theoretical Computations

**Figure 7:**
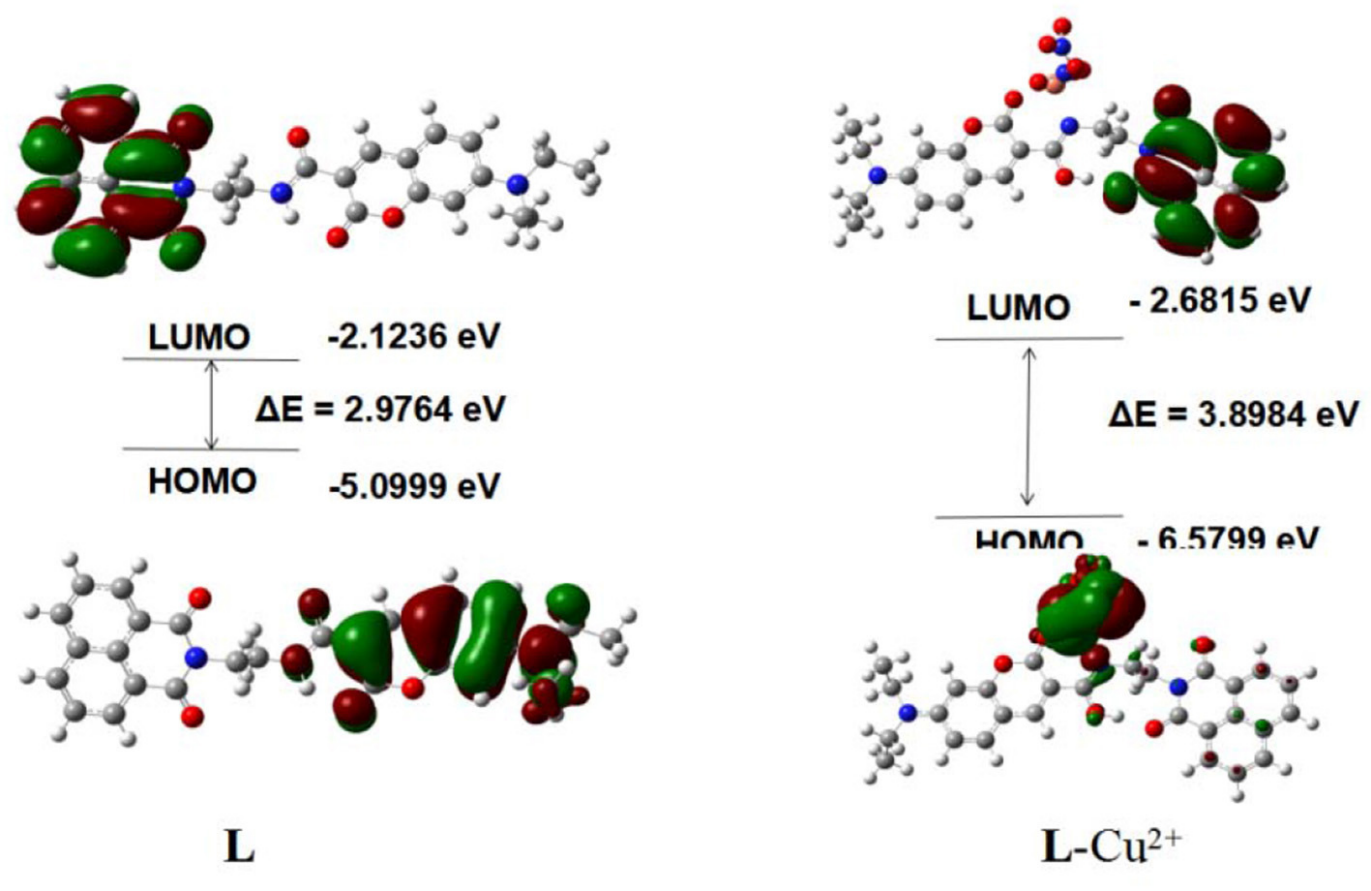
The results of **L** and **L**-Cu^2+^ by DFT.

To clarify the interaction mode between **L** and Cu^2+^, the orbital energy and spatial distribution levels of **L** and **L**-Cu^2+^ were gained with the DFT calculation (Figure 7). The electron density for **L** was mainly distributed over the coumarin groups in the highest occupied molecular orbital (HOMO), where the electron density for **L**-Cu^2+^ was focused on Cu^2+^ in the highest occupied molecular orbital (HOMO). The electron density was mainly located in the naphthalimide group in the lowest unoccupied molecular orbital (LUMO) of **L** and **L**-Cu^2+^. The energy gaps of the **L** and **L**-Cu^2+^ were calculated to be 2.9764 and 3.8984 eV, which were in accordance with the hypsochromic shift in the UV-Vis spectra after the Cu^2+^ solution was added into the **L** solution. The theoretical calculation results also confirmed the interaction mode between them.

### Application

**Figure 8:**
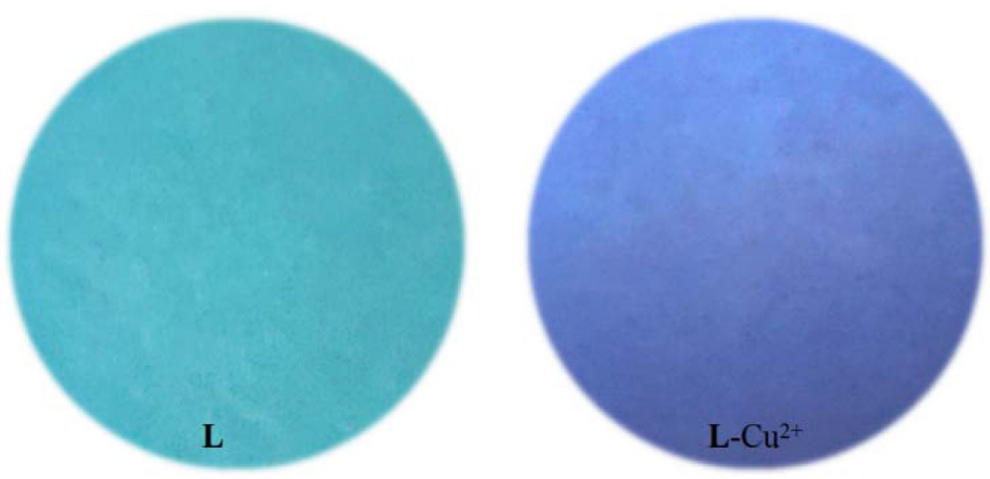
Photographs of **L** and **L**-Cu^2+^ (1 × 10^−5^ M) on test strips under a 365 nm UV lamp.

To evaluate the practical application of **L**, test strips were made from **L** to detect Cu^2+^, in which the filer paper was soaked in the **L** solution (1 × 10^−5^ M) and dried in the air. After the test strips were immersed in the Cu^2+^ solution (1.0 × 10^−5^ M), the test strips color change was examined directly by the naked-eye under a 365 UV lamp (Figure 8). It meant that probe **L** could also recognize Cu^2+^ in the solid state as well. In addition, test strips were made from **L** to detect Cu^2+^ in tap water (Supplementary Figure 9). It is interesting that only the aqueous solution containing the Cu^2+^ faded and the fluorescence decreased which shows that probe **L** could potentially identify Cu^2+^ water pollution.

**Figure 9:**
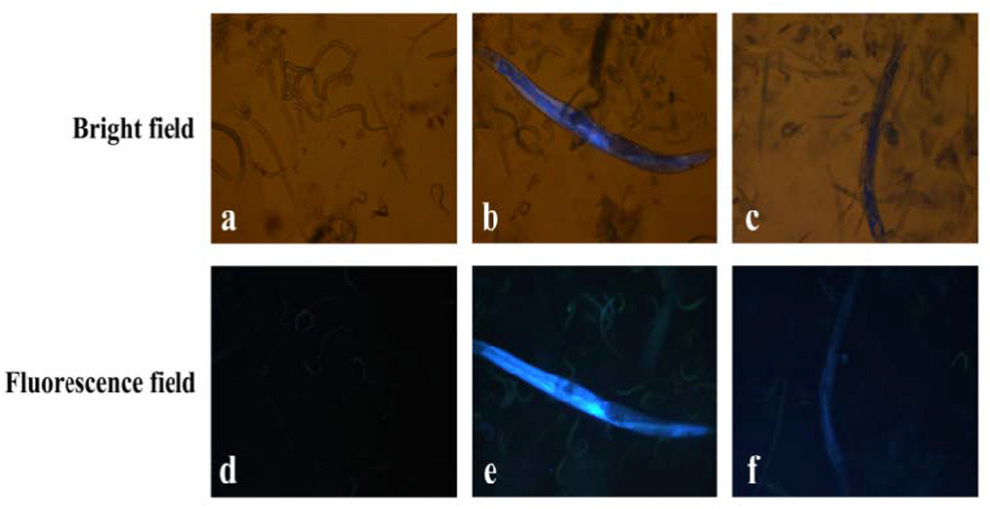
Bright field and fluorescent images of *C. elegans*: **(a,d)** *C. elegans* were incubated with agaragar for 30 min; **(b,e)** *C. elegans* were incubated with agaragar after pre-incubation with **L** (10 μM) for 30 min. **(c,f)** *C. elegans* were incubated with **L** (10 μM) for 30 min after pre-incubation with Cu^2+^ (10 μM) for 30 min.

To explore the application of **L** in the biological system, the ability of probe **L** to sense Cu^2+^ in adult *C. elegans* was studied (Figure 9). Bright field and fluorescent images of the *C. elegans* nematodes are shown in Figures 9A,D. The nematodes cultured with **L** exhibited blue fluorescence (Figures 9B,E). The fluorescence reduced obviously after the nematodes were cultured with Cu^2+^ (Figures 9C,E). This result showed the applicability of probe **L** to *in vivo* studies.

## Conclusion

In summary, probe **L**, based on naphthalimide-modified coumarin derivatives, was designed, synthesized, and characterized. Probe **L** showed good selectivity and high sensitivity toward Cu^2+^ while other metal ions did not cause interference. At the same time, the solution color change was observed directly by the naked-eye. The proposed interaction mode between them was confirmed by UV-Vis spectroscopy, fluorescence, Job's plot, ^1^H NMR titration, ESI-MS, and SEM. In addition, probe **L** has a good application prospect for detecting Cu^2+^ qualitatively. The LOD of **L** was 3.5 × 10^−6^ M. Additionally, a DFT calculation method was utilized to analyze the action mechanism of **L** toward Cu^2+^. Furthermore, the successful detection of Cu^2+^ in the living system using **L** also suggests its potential utilization in practical applications.

## Data Availability Statement

All datasets generated for this study are included in the article/Supplementary Material.

## Author Contributions

JZ and RQ designed the work and wrote the manuscript. JZ, M-YC, BW, LZ, and C-QQ carried out the experiments. R-QL performed the spectroscopic experiments. C-BB revised and edited the manuscript. All authors reviewed the manuscript and have agreed to its publication.

## Conflict of Interest

The authors declare that the research was conducted in the absence of any commercial or financial relationships that could be construed as a potential conflict of interest.
