# Supplementary material for: A Coumarin-Based Fluorescent Probe for Ratiometric Detection of Cu2+ and Its Application in Bioimaging
Source: Front Chem. 2020 Oct 2;8:800. doi: 10.3389/fchem.2020.00800 (PMC7573568; doi:10.3389/fchem.2020.00800)
Supplement: Supplementary file 1 [file Table_2.DOC]

Supplementary Material

A Coumarin-Based Fluorescent Probe for Ratiometric Detection of Cu^2+^ and Its Application in Bioimaging

**Jie Zhang^1^, Meng-Yu Chen^1^, Cui-Bing Bai****^1, 2, 4*^, Rui Qiao^1, 2, 4*^, Biao Wei^1, 4^, Lin Zhang^1, 4^, Rui-Qian Li^1, 4^, and Chang-Qing Qu^3*^**

^1^School of Chemistry and Materials Engineering, Fuyang Normal University, Fuyang, Anhui, 236037, P. R. China

^2^Key Laboratory of Photochemical Conversion and Optoelectronic Materials, TIPC, Chinese Academy of Sciences, Beijing, 100190, P. R. China

^3^Research Center of Anti-aging Chinese Herbal Medicine of Anhui Province, Fuyang, Anhui, 236037, P. R. China

^4^Engineering Research Center of Biomass Conversion and Pollution Prevention of Anhui Educational Institutions, Fuyang, Anhui, 236037, P. R. China

E-mail: [baicuibing@fynu.edu.cn (C.B.B.);](mailto:baicuibing@126.com;) [qiaorui@](mailto:qiaorui@mail.ipc.ac.cn)fynu.edu.cn (R.Q.); [qucq518@163.com](mailto:qucq518@163.com) (C.Q.Q.)


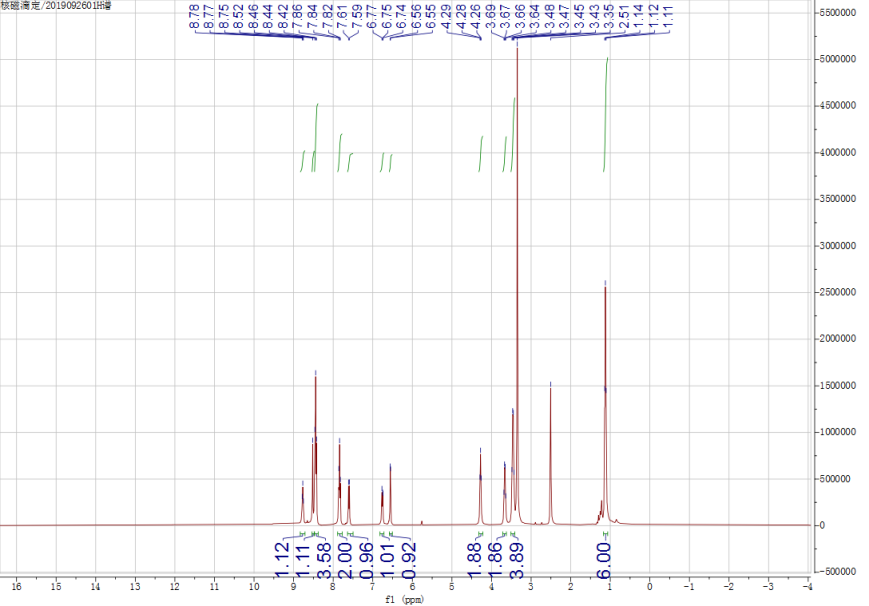


**Figure S1.** ^1^H NMR spectrum of compound **L** in *d_6_*-DMSO.


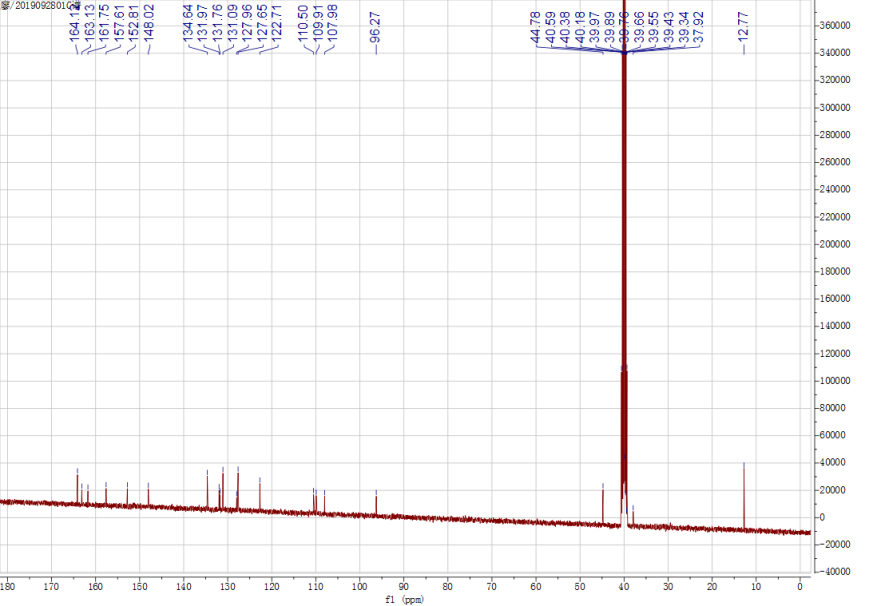


**Figure S2.** ^13^C NMR spectrum of compound **L** in d*_6_*-DMSO.

(a)


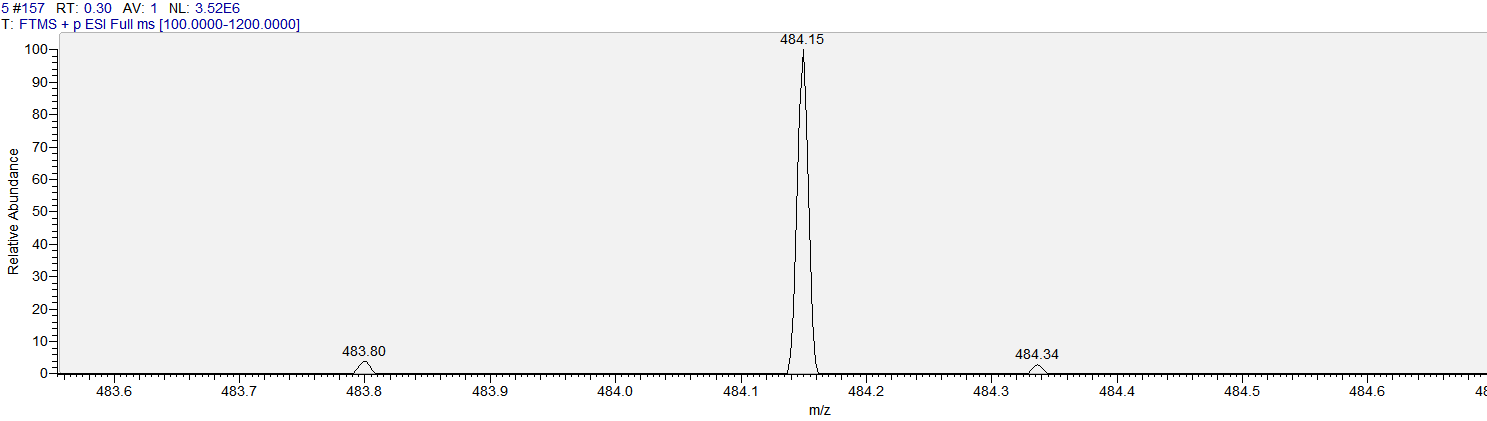


(b)

**
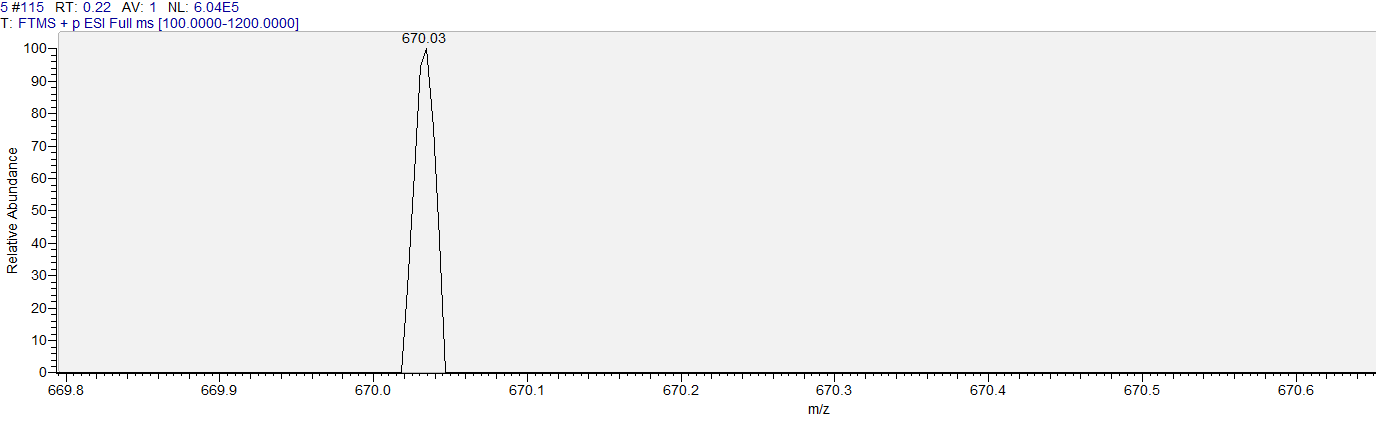
**

**Figure S3.** (a) ESI-MS spectrum of [**L**+H] ^+^, (b) ESI-MS spectrum of [**L**+Cu^2+^+2NO_3_^-^] ^+^ complex

(a)

**
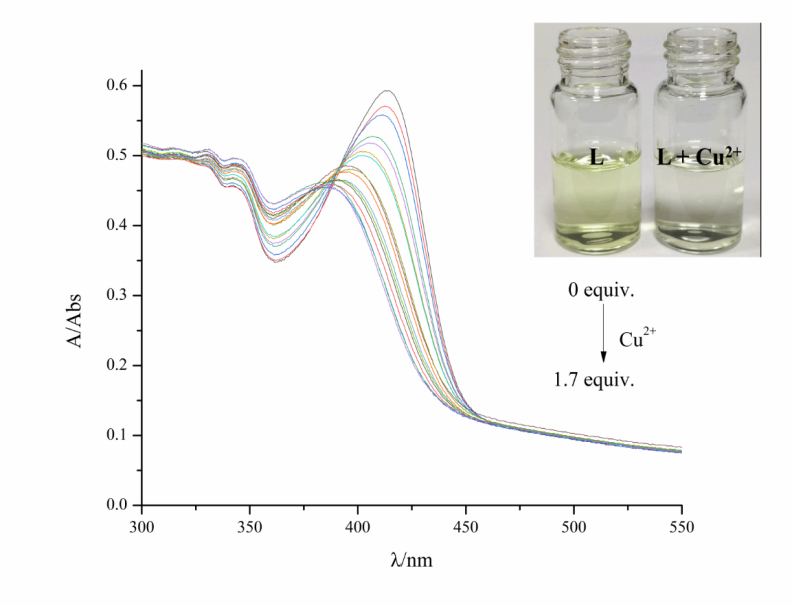
**

(b)

**
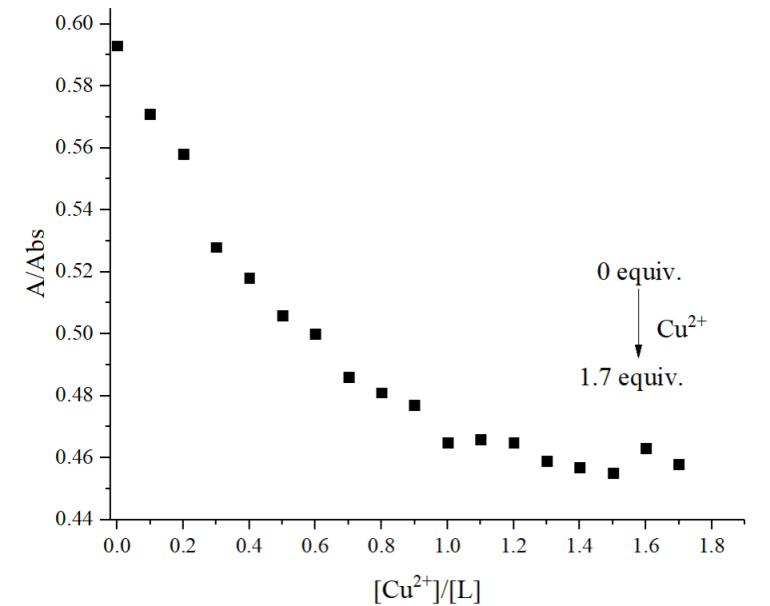
**

**Figure S4.** (a) Absorption spectrum of **L** (1.0×10^-5^ M) in the presence of different concentration of Cu^2+^ (0-1.7 equiv.) in HEPES buffer (10 mM, pH=7.4)/CH_3_CN (1:4, V/V). (b) A plot of absorption at 412 nm depending on the concentration of Cu^2+^ in the range from 0 to 1.7 equiv.

(a)


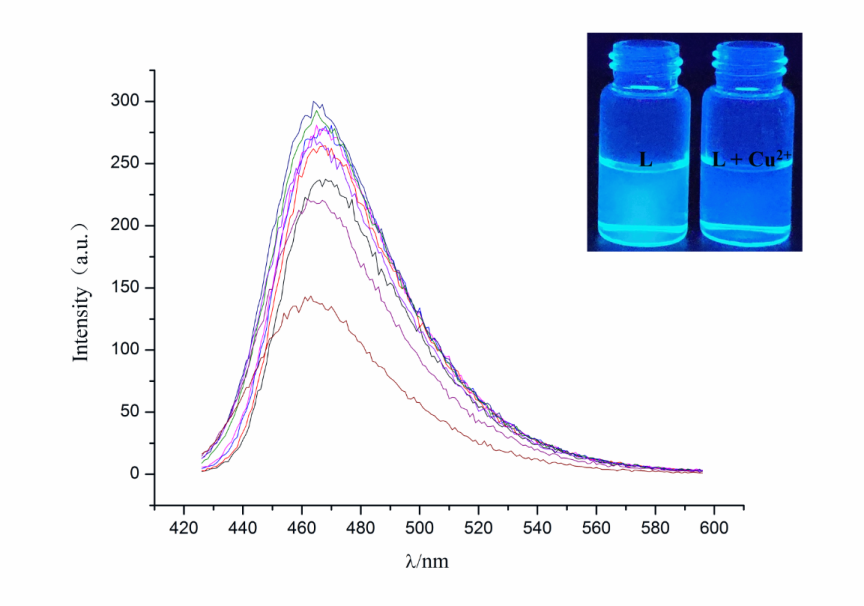


(b)

**
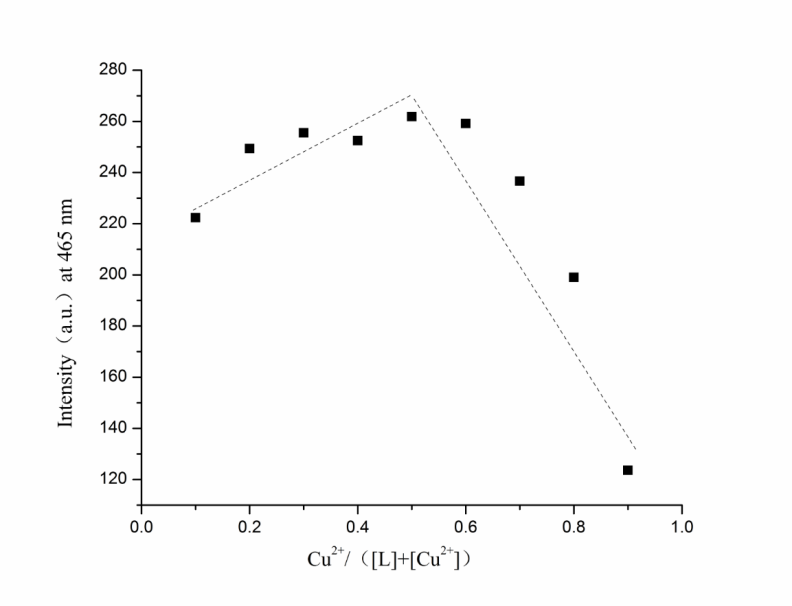
**

**Figure S5.** (a) Fluorescence spectrum of probe **L** in the presence of Cu^2+^ with different mole ratios of [Cu^2+^]/ ([Cu^2+^+**L**]) at the constant total concentration ([Cu^2+^] + [**L**]) = (2.0 × 10^-5^ M) in HEPES buffer (10 mM, pH=7.4)/CH_3_CN (1:4, V/V), λ_ex_= 412 nm, at 465 nm. (b) Job’s plot for determination of the binding stoichiometry of probe **L** with Cu^2+^.

**
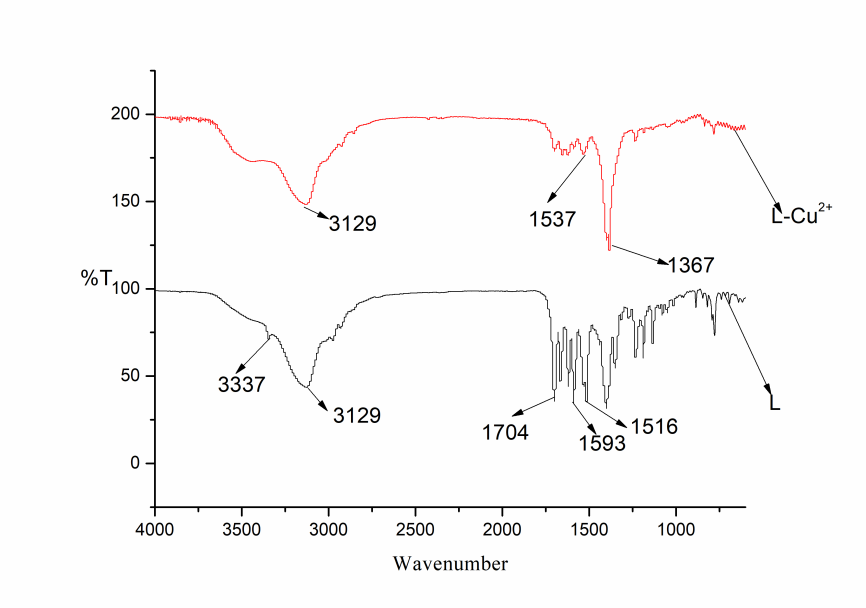
**

**Figure S6.** The FT-IR spectrum for **L** and **L**-Cu^2+^

**
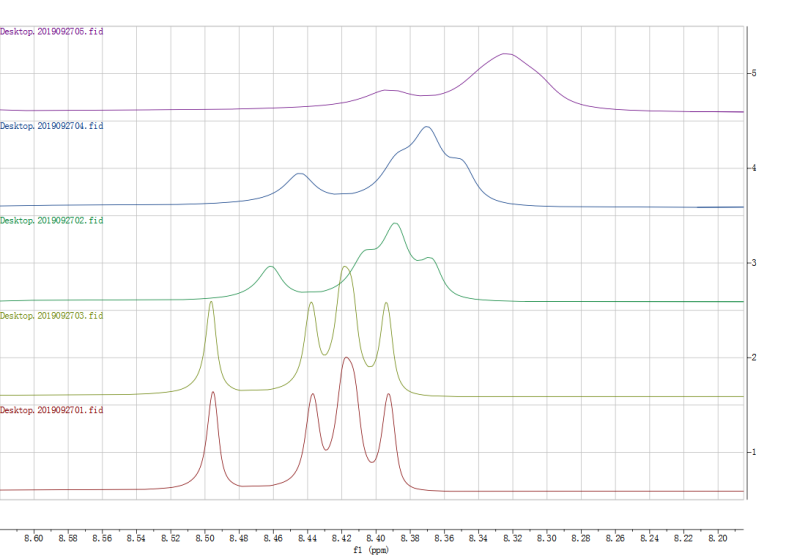
**

**Figure S7.** Chemical shift change of adding different proportion of Cu^2+^ from low to high (0.3 equiv, 0.5 equiv, 0.7 equiv, 1.0 equiv, 1.3 equiv).


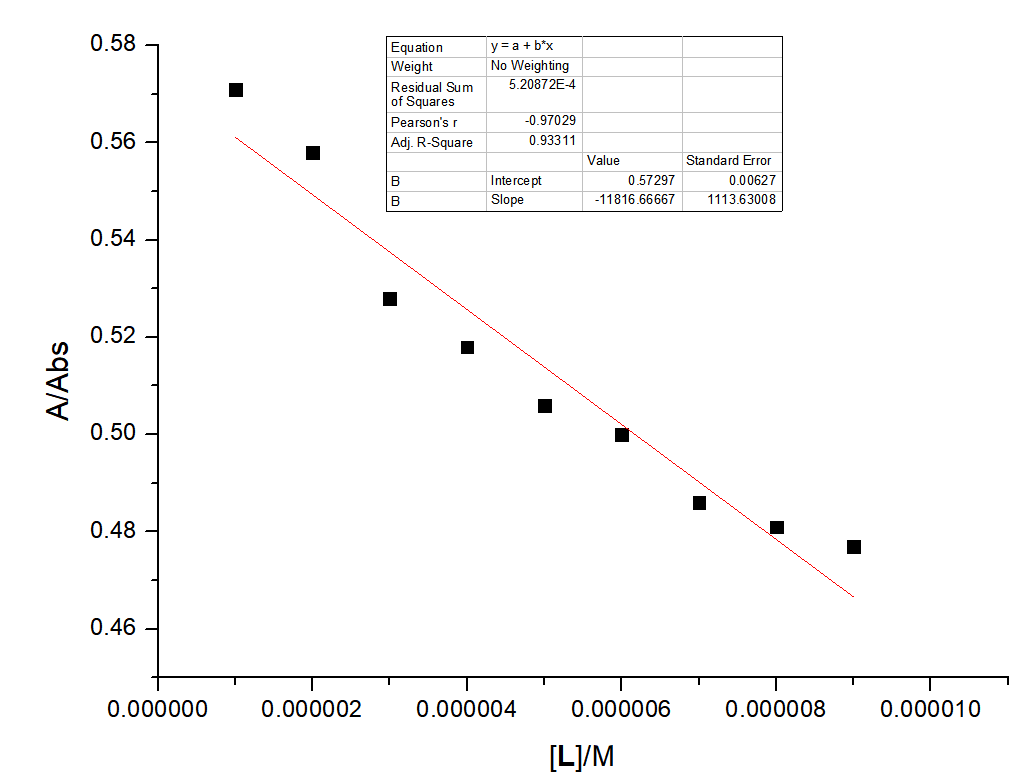


**Figure S8.** Detection limit of **L** (1.0×10^-5^ M) towards the detection of Cu^2+^.

The detection limits of **L** towards Cu^2+^ were determined from the following equation: DL=3•SD/S

S = 11816.66667

δ =$\sqrt{\frac{\sum\left(A_{0}-A_{1}\right)^{2}}{\left(N-1\right)}}$ = 0.0138 (N = 20), K = 3

LOD = K × δ / S = 3.5 × 10^−6^ M

Where SD is the standard deviation of the blank solution (**L**) detected for 20 times; S is the slope of the calibration curve. A_0_ is the absorption intensity of **L**; A_1_ is the average of the A_0_.

(a)


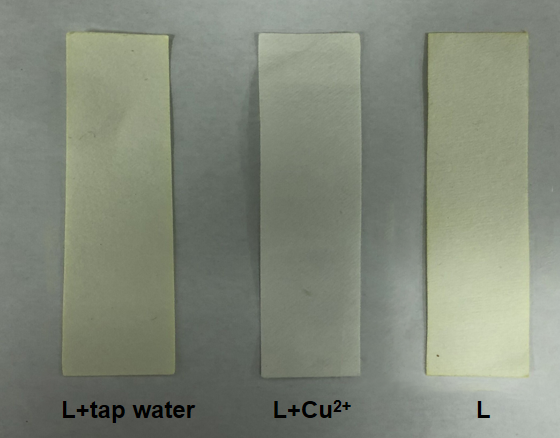


(b)


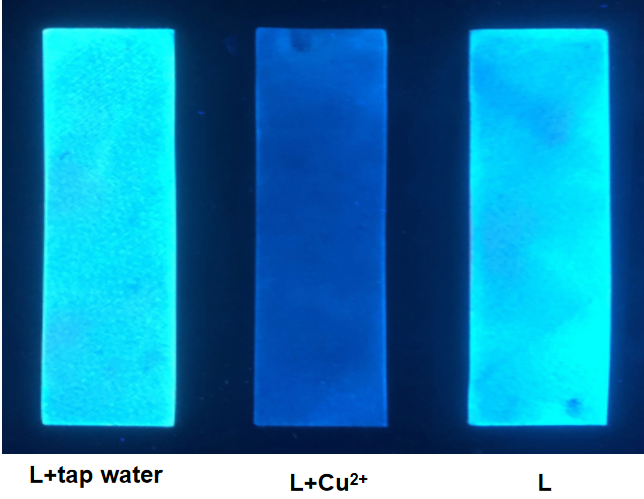


**Figure S9.** Test papers immersed in tap water contaminated with Cu^2+^ contaminants, from

left to right: **L**+tap water and tap water of Cu^2+^+**L**, and **L** (1×10^-4^ M).


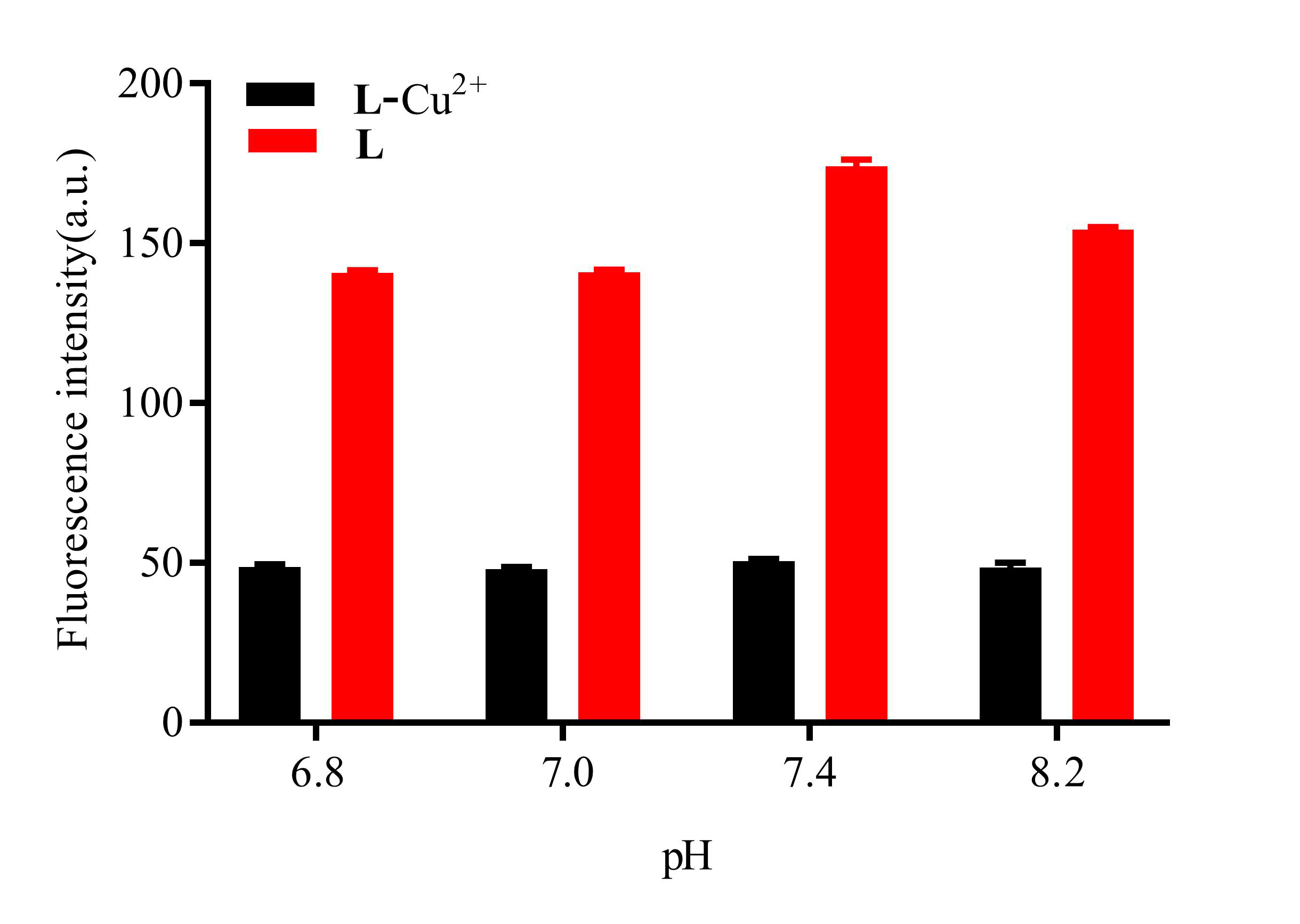


**Figure S10.** The pH (6-8) of **L** (1.0×10^-5^ M) and pH of **L**-Cu^2+^.
