# Supplementary material for: A Coumarin-Based Fluorescent Probe for Ratiometric Detection of Cu2+ and Its Application in Bioimaging
Source: Front Chem. 2020 Oct 2;8:800. doi: 10.3389/fchem.2020.00800 (PMC7573568; doi:10.3389/fchem.2020.00800)

**A Coumarin-Based Fluorescent Probe for Ratiometric Detection of Cu^2+^ and Its Application in Bioimaging**

**Jie Zhang^1^, Meng-Yu Chen^1^, Cui-Bing Bai****^1, 2, 4*^, Rui Qiao^1, 2, 4*^, Biao Wei^1, 4^, Lin Zhang^1, 4^, Rui-Qian Li^1, 4^, and Chang-Qing Qu^3*^**

^1^School of Chemistry and Materials Engineering, Fuyang Normal University, Fuyang, Anhui, 236037, P. R. China

^2^Key Laboratory of Photochemical Conversion and Optoelectronic Materials, TIPC, Chinese Academy of Sciences, Beijing, 100190, P. R. China

^3^Research Center of Anti-aging Chinese Herbal Medicine of Anhui Province, Fuyang, Anhui, 236037, P. R. China

^4^Engineering Research Center of Biomass Conversion and Pollution Prevention of Anhui Educational Institutions, Fuyang, Anhui, 236037, P. R. China


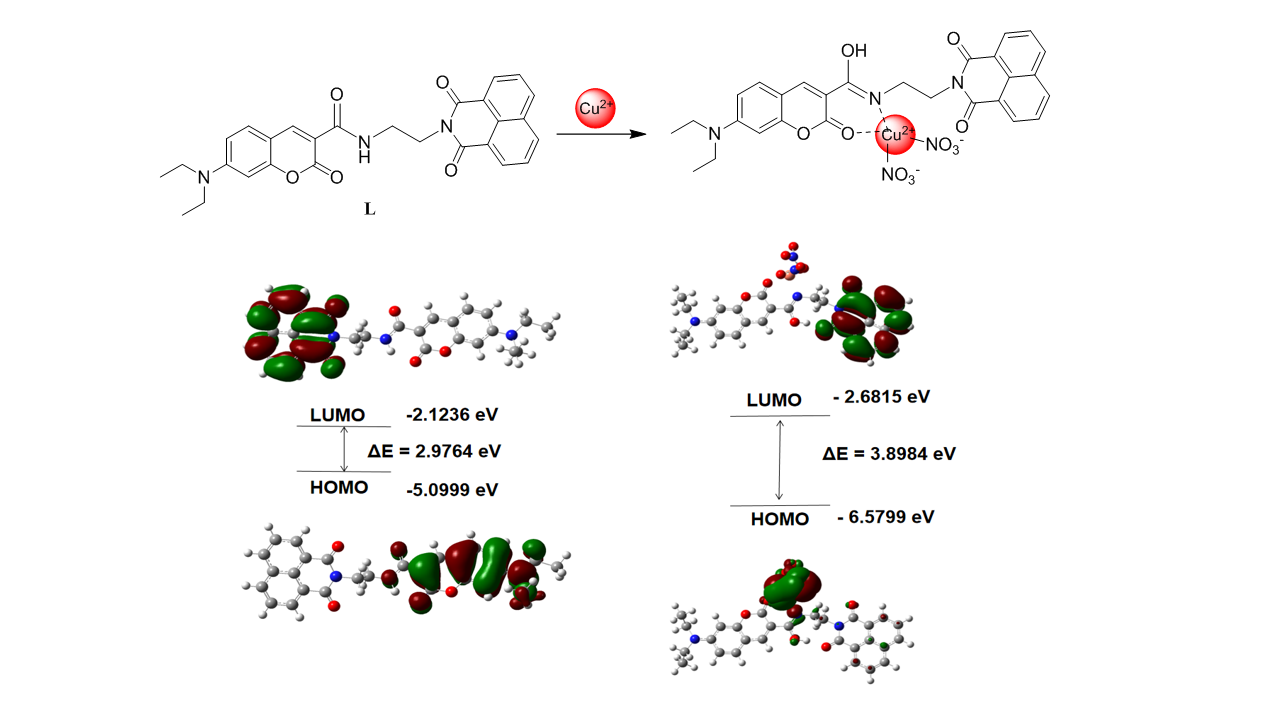

Supplement: Supplementary file 2 [file Table_1.DOC]
